# Transthyretin as a novel candidate biomarker for preeclampsia

**DOI:** 10.3892/etm.2014.1558

**Published:** 2014-02-18

**Authors:** LEI ZHU, YUXUAN CHEN, CHONGDONG LIU, HAITENG DENG, NAWEI ZHANG, SHENGDIAN WANG, ZHENYU ZHANG

**Affiliations:** 1 Department of Obstetrics and Gynecology, Beijing Chaoyang Hospital Affiliated to Capital Medical University, Beijing 100020, P.R. China; 2 The Rockefeller University, New York, NY 10065, USA; 3 Institute of Biophysics, Chinese Academy of Sciences, Beijing 100101, P.R. China

**Keywords:** serum biomarker, preeclampsia, transthyretin

## Abstract

Preeclampsia (PE) is considered to be a potentially fatal complication during pregnancy. However, no effective laboratory assessment has been developed to enable early diagnosis and monitoring of PE. The present study aimed to identify differentially expressed transthyretin (TTR) during severe PE and evaluate TTR as a possible biomarker of this disease. TTR levels were determined in the different gestational weeks of normal pregnancy (before 20 weeks, n=41; after 20 weeks, n=39) using enzyme-linked immunosorbent assay (ELISA). TTR concentrations in pregnant females with severe PE (n=43) were compared with those in healthy matched control subjects (n=37) using western blot analysis and ELISA. The median TTR concentration during severe PE in each month of gestation was significantly lower than the concentrations recorded during normal pregnancy. TTR levels in females with severe PE were significantly downregulated compared with the control subjects (P<0.001; area under the curve, 0.834–0.967). Thus, TTR may be used as a potential biomarker of PE.

## Introduction

Preeclampsia (PE) is a multisystem syndrome affecting pregnant females. PE usually develops after 20 weeks of gestation and affects 4–10% of pregnant females. PE is characterized by several symptoms, including hypertension, proteinuria and additional complications such as liver and kidney failure and fetal distress. Approximately 25% of babies born to females with PE are smaller than normal for the particular gestational age. PE is a predominant cause of maternal morbidity and mortality worldwide (1,2). Although the exact determinants of PE remain unclear, placental ischemia is considered to be important in the development. The hypoxic placenta may lead to an imbalance in the release of circulating factors, which may result in widespread vascular endothelial injury. Certain proteomic factors, including antiangiogenic factors, may contribute to systemic hypertension, vascular injury and disorders of the coagulation system (3–5). Variations in these circulating proteomic factors have been shown to correlate with pathophysiological changes in the disease.

Early diagnosis of PE is reliant upon the provision of regular antenatal care prior to delivery. To date, no biomarker-based laboratory assessment is able to diagnose PE. Investigations have been conducted to identify non-invasive, blood-borne or urinary maternal biomarkers that predict the development of PE and aid in the monitoring of this severe complication during pregnancy (1,6). Potential biochemical markers, including soluble fms-like tyrosine kinase 1 (sflt-1) and placental growth factor (PLGF), have been identified, however are not considered to be reliable in the diagnosis of PE (1,5,6). Therefore, identification of effective markers is required to predict PE.

In a previous study, serum proteomic analysis of PE was performed, revealing decreased transthyretin (TTR) concentrations in the sera of females with PE (7). TTR is a tetrameric serum protein composed of four identical subunits (55 kDa) and is predominantly synthesized in the liver, eye and choroid plexus. A protein group comprising TTR, thyroxin-binding globulin and albumin, bind to and transport thyroid hormones in the blood; the main function of TTR is the transport of thyroxin (T_4_) (8). TTR is synthesized by placental trophoblasts which are critical to normal fetal development. Thus, disorders caused by TTR production may result in fetal distress (9–11). In addition, >100 TTR mutations have been shown to be associated with amyloid diseases, which induce tissue-selective deposition of amyloid to various organs (12,13). In a previous study, TTR was shown to be upregulated by two-fold in pancreatic cancer, thus, it was concluded that TTR may be used as a novel tumor marker (14). However, whether TTR may be used as a biomarker of PE remains unknown.

In the present study, significant changes in TTR expression levels during severe PE were observed. It was hypothesized that the differences in TTR concentrations during severe PE were associated with disease pathophysiology, thus, TTR may be a candidate biomarker of PE.

## Materials and methods

### Grouping

Three experiments were conducted to identify the changes in TTR levels during severe PE. Changes in TTR levels during healthy pregnancy were observed as follows: A series of samples were collected from normal pregnant females at different gestation periods to identify the TTR concentrations during healthy gestation (before 20 weeks, n=41; after 20 weeks, n=39). TTR levels in females with severe PE were compared with the levels of those in the normal control subject group. A total of 43 females after 20 weeks of gestation were selected as participants in the severe PE group; these females were free of other pregnancy complications. No subjects had a history of hypertension or renal disease. A total of 37 healthy females were enrolled in the control group and matched to the females in the severe PE group with regard to gestational age. TTR levels in the severe PE and control groups were monitored simultaneously. TTR levels in the early (n=21) and late (n=22) onset PE patients were compared (all of these cases were included in the severe PE group). The characteristics of participants are presented in Tables I–III. The serum samples were all collected from Chinese females.

Severe PE is defined as an increase in blood pressure (≥160 mmHg systolic pressure or ≥110 mmHg diastolic pressure on more than two occasions at an interval of at least 6 h) that occurs following 20 weeks of gestation in females with normal blood pressure, accompanied by proteinuria (serum protein ≥5 g/24 h or ≥2^+^ calculated via dipstick measurement), coagulopathy disorders (platelets <100×10^9^/l or disseminated intravascular coagulation), liver dysfunction and acute renal disorders.

### Sample collection

Samples were collected from the peripheral blood and prepared by centrifugation at 2,415 × g for 10 min at 4°C within 4 h following acquisition. Samples were subsequently stored at −80°C until use. The samples were collected from the Department of Obstetrics and Gynecology of Beijing Chaoyang Hospital affiliated to Capital Medical University (Beijing, China). This study was approved by the Ethics Committee of Beijing Chaoyang Hospital affiliated to Capital Medical University and informed consent was provided by each participant.

### Western blot analysis

TTR expression levels in severe PE and healthy control subjects were evaluated by western blot analysis (severe PE, n=43; control subjects, n=37). Total serum protein concentrations were determined using the bicinchoninic acid assay method (BCA Protein Assay Reagent; Thermo, Rockford, IL, USA). Samples of 60 μg serum protein from the two groups were run on 15% sodium dodecyl sulfate-polyacrylamide gel electrophoresis. The proteins were transferred to nitrocellulose membranes (Millipore Corporation, Billerica, MA, USA) and subjected to 300 mV for 25 min. The membranes were blocked overnight at 4°C in blocking buffer (containing 5% skimmed milk, 0.1% Tween 20 and 0.01 M tris-buffered saline) and incubated with primary antibodies against TTR (mouse monoclonal antibody, 1:1,000 dilution; Abcam, Cambridge, UK) for 120 min at room temperature. The membranes were incubated with goat anti-mouse IgG secondary antibody (Santa Cruz Biotechnology, Inc., Dallas, TX, USA) for 50 min. Protein levels were analyzed by evaluation of the total signal intensities of the western blot bands.

### Identification of TTR levels using an enzyme-linked immunosorbent assay (ELISA)

Serum TTR levels were determined by ELISA (Assaypro, St. Charles, MO, USA). Samples were diluted for detection (1:40,000)and the data were presented using CurveExpert 1.3.

### Statistical analysis

Data were analyzed using SPSS 17.0 software (SPSS, Inc, Chicago, IL, USA) with the independent samples t-test. P<0.05 was considered to indicate a statistically significant difference. The diagnostic value of TTR for severe PE was determined based on receiver operating characteristic (ROC) curves which were analyzed using MedCalc 9.6.2.0 (MedCalc Software bvba, Ostend, Belgium).

## Results

### Detection of TTR concentrations during healthy pregnancy

**Figure 1:**
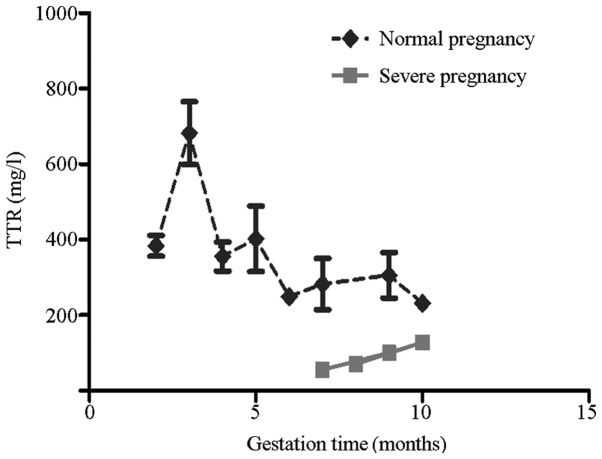
Comparison between TTR concentrations in the severe PE and healthy pregnancy groups. Each point on the graph indicates the median value per gestation month. In the normal pregnancy group, TTR levels were highest in the third month (9–12 weeks) of gestation and decreased markedly in the fifth month. No significant difference was identified in the second and third trimester. In the severe PE group (median TTR concentrations per gestation month following 20 weeks of gestation are shown), TTR concentrations significantly decreased compared with the healthy pregnancy group at the equivalent gestation time (P<0.001). TTR, transthyretin; PE, preeclampsia.

TTR concentrations in the three trimesters of normal pregnancy were determined using ELISA kits (Fig. 1). TTR levels significantly increased in the third month of gestation and rapidly decreased following 20 weeks of gestation. TTR levels in the initial 20 weeks of gestation were considerably higher compared with those following 20 weeks of gestation (P<0.001). No further changes in TTR levels were observed thereafter.

### Western blot analysis of TTR changes during severe PE

**Figure 2:**
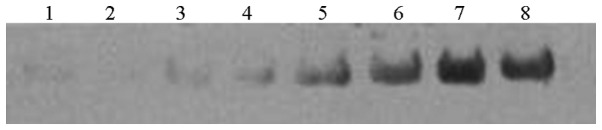
Western blot analysis results showing TTR concentrations in the sera of patients in the severe PE and control groups. TTR levels significantly decreased in the severe PE group (P<0.001). Lanes 1–4, severe PE group; lanes 5–8, control group. TTR, transthyretin; PE, preeclampsia.

TTR expression levels were detected by western blot analysis to directly monitor the changes in TTR during severe PE. A total of 43 females with severe PE and 37 control subjects were enrolled in the present study. Fig. 2 shows the expression levels of TTR in the sera of the two groups. The single band at ~16 kDa represented the TTR monomer undergoing dissociation. TTR expression levels were markedly decreased in the sera of patients with severe PE and were ~2.6 times lower compared with the control subjects (4,867±3,464 vs. 12,517±8,516 OD units in the severe PE and control subjects, respectively; P<0.001).

### Identification of TTR levels during severe PE using ELISA

**Figure 3:**
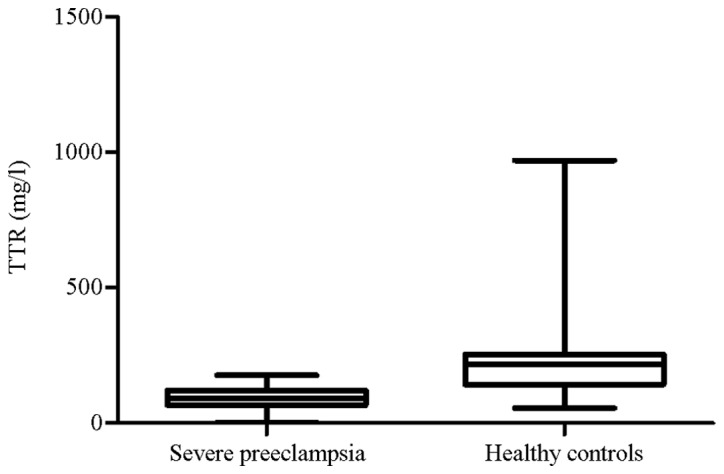
Distribution of TTR levels in the severe PE and control groups. The horizontal line indicates the median value per group. Ends of the columnar graph indicate the highest and lowest values per group. TTR, transthyretin; PE, preeclampsia.

ELISA analysis was conducted to quantify the TTR levels of the patients in the severe PE (n=43) and control (n=37) groups. TTR levels significantly decreased in the severe PE group (P<0.001) and were ~2.4 times lower compared with the control group. This result is consistent with those of the western blot analysis. The median TTR concentration of the severe PE group was significantly lower compared with the control group (Fig. 3). In Fig. 1, the median TTR concentrations in the severe PE and healthy subjects at the same gestation period were compared. The curve for the severe PE group was significantly lower than that for the healthy pregnancy group.

### TTR levels in early and late onset PE

Among the 43 participants in the severe PE group, 21 individuals were assigned to the early onset group and 22 individuals were assigned to the late onset group. TTR levels were lower in the early onset patients than in the late onset group (P<0.001).

### Diagnostic value of TTR for severe PE

**Figure 4:**
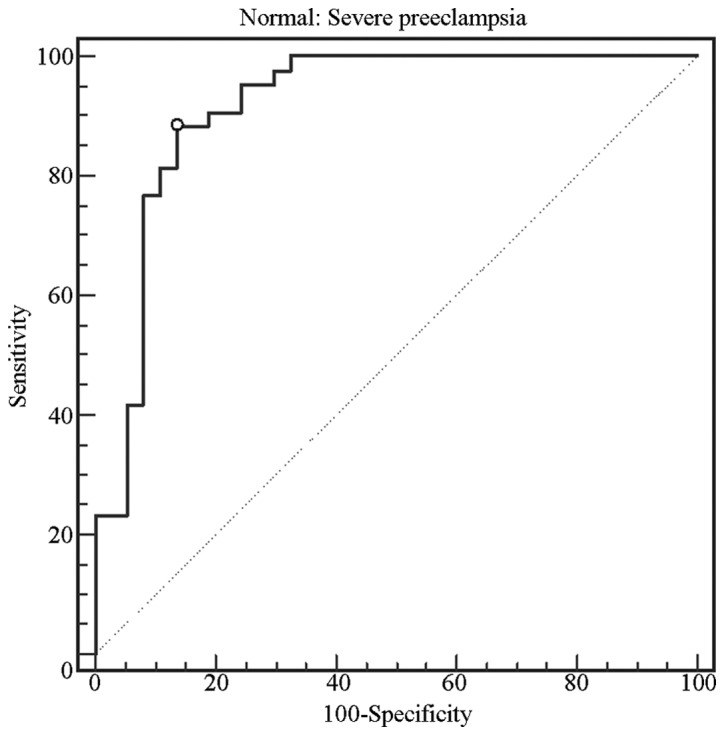
ROC curve for TTR. Area under the curve, 0.917, range, 0.834–0.967. TTR, transthyretin; ROC, receiver operating characteristic.

The diagnostic value of TTR in severe PE pregnancies was examined with ROC curves (Fig. 4). The results indicated that TTR may be a reliable biomarker for the diagnosis of severe PE, exhibiting sensitivity and specificity levels of 88.4 and 86.5%, respectively (area under the curve, 0.917, range, 0.834–0.967), at a diagnostic value of 128.81 mg/l.

## Discussion

The identification of novel and effective biomarkers of PE is critical for early prediction, prognosis, monitoring and treatment responses. Angiogenic factors, including sflt-1 and PLGF, have been considered as potential biomarkers of PE. However, previous studies have presented inconsistencies with regard to the use of various potential markers to diagnose PE. Therefore, further studies are required to identify and develop effective and efficient biomarkers of PE (1,15).

ELISA analysis results of healthy pregnant subjects identified that TTR concentrations rapidly increased in the third month of pregnancy (9–12 weeks). TTR levels were higher prior to 20 weeks of gestation. Administration of maternal thyroid hormone to facilitate fetal development contributes to the rapid increase in TTR levels in the early stages of gestation (10,11,16). A previous study identified that fetuses are unable to synthesize TTR prior to 16 weeks of gestation. In addition, Fig. 3 shows that the highest TTR levels were observed during the third month (9–12 weeks). Lower TTR levels were observed following the fifth month (17–20 weeks) of gestation. Therefore, this result is consistent with results of a previous study, which indicated that maternal TTR proteins may be important in transporting thyroid hormone to the fetus and may be required for fetal development (17). Lower cord serum levels of T_4_ in preterm infants have been shown to correlate with gestation week and weight. In contrast to term infants, preterm infants frequently experience a decrease in serum T_4_ levels, which may account for the increased rate of morbidity and mortality (18). These results indicate that the thyroid gland of the fetus does not provide adequate quantities of T_4_ prior to full term delivery and the maternal thyroid hormone compensates for such insufficient T_4_ levels during fetal development. Maternal TTR, as a transporter of thyroid hormone, functions in transporting T_4_ to the fetus (11). Although fetuses may produce TTR proteins, maternal TTR protein may be important in fetal development.

The present study revealed that TTR levels were significantly decreased in the sera of females with severe PE. This result was consistent with that of a previous proteomic analysis, in which decreased levels of TTR were observed in early onset cases of severe PE (19). The median maternal TTR levels were significantly lower in the severe PE group than in the control group. In addition, the curve for the severe PE group was markedly lower compared with the control group in the equivalent gestation month, indicating that the changes in TTR levels during severe PE may correlate with disease development. ROC curves were used to evaluate the reliability of TTR as a diagnostic tool for severe PE (Fig. 4). TTR levels were capable of discriminating between severe PE and healthy females (sensitivity and specificity of 88.4 and 86.5%, respectively) at a cutoff value of 128.81 mg/l.

TTR concentrations in early and late onset cases of severe PE were compared. Diagnosis at earlier gestational stages has been reported to indicate a higher risk of maternal and fetal mortality (20,21). In the present study, TTR levels were markedly decreased in early onset severe PE cases, indicating that the changes in TTR levels may correlate with the severity of PE. Furthermore, these changes may be used to monitor severe complications.

In the present study, two hypotheses were presented to explain the changes in TTR levels during severe PE. Initially, it was hypothesized that the decreased TTR levels may contribute to the pathology of PE. Maternal vascular dysfunction, that induces multi-organ disorders, was considered to be the basic pathological manifestation of PE (3,4,22). The TTR tetramer dissociates to produce a non-native TTR monomer with low conformational stability, thereby forming TTR amyloids, which bind to the vascular wall and lead to changes in membrane fluidity (23–25). Therefore, TTR may damage the maternal vascular system via amyloid deposition and this condition may be attributed to the decreased TTR levels during severe PE. TTR amyloid fibrils may be selectively deposited in the maternal vascular system, resulting in organ ischemia of the placenta, liver, kidney and brain, as well as other clinical manifestations (26–29). Therefore, TTR concentrations may change prior to the onset of PE and may be used as a potential biomarker to predict and monitor PE. The second hypothesis suggested that the changes in the TTR levels of the severe PE group may have resulted from reduced production of the TTR protein. Previous studies have identified that females with PE exhibit a greater risk of subclinical hypothyroidism during pregnancy, which is attributed to vascular endothelial growth factor inhibitors that damage the endothelium of thyroid capillaries; subclinical hypothyroidism is also involved in reducing the production of thyroid hormone (30). Decreased TTR expression may also be responsible for vascular injury of the placenta. TTR secreted from the placenta is involved in the transport of the maternal thyroid hormone into the fetal circulation via the TTR-T_4_ complex, which is important in fetal development (10,11,18). As the predominant pathological mechanism of changes in PE, placenta necrosis may result in decreased TTR secretion and lead to disorders during fetal development under severe PE conditions. If the 2nd hypothesis is correct, the decreased level of TTR should be a result of PE pathological variations and may be it is not lower than normal before PE is diagnosed and may not be able to be a predictor of PE. However, hormonal secretion by the placenta may be impaired by continued placenta necrosis, resulting in a further decrease in TTR expression in maternal serum. Therefore, lower maternal TTR levels may indicate that PE has worsened.

In conclusion, the present study has revealed that TTR levels are significantly decreased in severe PE and may be associated with the various changes observed during PE. Therefore, TTR may be used as a candidate biomarker of PE. However, further studies are required to confirm whether TTR functions in the pathology of PE and whether TTR levels change during mild PE. Thus, changes in TTR concentrations prior to the onset of PE require further investigation.

## Figures and Tables

**Table I:** Characteristics of the severe PE and control groups.

Characteristics	Control (n=37)	Severe PE (n=43)
Age, years	27±3^a^	28±4
Gestation length, weeks (range)	33±4^b^ (25–38)	33±3 (25–37)
Systolic MAP, mmHg	115±10	159±18
Diastolic MAP, mmHg	71±9	99±13
Proteinuria, g/24 h	Negative	5±1
Placenta weight, g	692±135^c^	623±125
Infant birth weight, g	3,245±525^d^	1,917±532

Age, gestation length and blood pressure are expressed as the mean ± SD. No significant difference was identified between the severe PE and control groups:

a P=−0.274 and

b P=0.983, respectively. Placenta weight and infant weight were significantly lower in the severe PE group:

c P=0.023 and

d P=0.005, respectively.

PE, preeclampsia; MAP, mean arterial pressure.

**Table II:** Characteristics of the healthy pregnancy group.

Characteristics	Before 20 weeks of gestation (n=41)	After 20 weeks of gestation (n=39)
Age, years	28±4^a^	28±4
Systolic MAP, mmHg	118±11	116±15
Diastolic MAP, mmHg	72±8	75±10
Proteinuria, g/24 h	Negative	Negative

a P=0.344, no significant difference was identified between the groups.

MAP, mean arterial pressure.

**Table III:** Characteristics of early and late onset PE.

Characteristics	Early onset PE (n=21)	Late onset PE (n=22)
Systolic MAP, mmHg	160±13	155±15
Diastolic MAP, mmHg	112±10	92±15
Proteinuria, g/24 h	6±1	5±0
Placenta weight, g	603±123^a^	645±130
Infant birth weight, g	1,489±542^b^	2,200±447

a P=0.045 and

b P=0.002.

Placenta and infant weights were lower in early onset PE patients. PE, preeclampsia; MAP, mean arterial pressure.
